# Genetic Association between Swine Leukocyte antigen Class II Haplotypes and Reproduction Traits in Microminipigs

**DOI:** 10.3390/cells8080783

**Published:** 2019-07-26

**Authors:** Asako Ando, Noriaki Imaeda, Tatsuya Matsubara, Masaki Takasu, Asuka Miyamoto, Shino Oshima, Naohito Nishii, Yoshie Kametani, Takashi Shiina, Jerzy K. Kulski, Hitoshi Kitagawa

**Affiliations:** 1 Department of Molecular Life Science, Division of Basic Medical Science and Molecular Medicine, Tokai University School of Medicine, Isehara 259-1193, Japan; 2 Department of Veterinary Medicine, Faculty of Applied Biological Sciences, Gifu University, Gifu 501-1193, Japan; 3 Faculty of Health and Medical Sciences, UWA Medical School, The University of Western Australia, Crawley WA 6009, Australia; 4 Laboratory of Veterinary Internal Medicine, Faculty of Veterinary Medicine, Okayama University of Science, 1-3 Ikoino-oka, Imabari, Ehime 794-8555, Japan

**Keywords:** swine leukocyte antigen, reproductive performance, production trait, haplotype, micro-mini-pigs

## Abstract

The effects of swine leukocyte antigen (SLA) molecules on numerous production and reproduction performance traits have been mainly reported as associations with specific SLA haplotypes that were assigned using serological typing methods. In this study, we intended to clarify the association between SLA class II genes and reproductive traits in a highly inbred population of 187 Microminipigs (MMP), that have eight different types of SLA class II haplotypes. In doing so, we compared the reproductive performances, such as fertility index, gestation period, litter size, and number of stillbirth among SLA class II low resolution haplotypes (Lrs) that were assigned by a polymerase chain reaction-sequence specific primers (PCR-SSP) typing method. Only low resolution haplotypes were used in this study because the eight SLA class II high-resolution haplotypes had been assigned to the 14 parents or the progenitors of the highly inbred MMP herd in a previous publication. The fertility index of dams with Lr-0.13 was significantly lower than that of dams with Lr-0.16, Lr-0.17, Lr-0.18, or Lr-0.37. Dams with Lr-0.23 had significantly smaller litter size at birth than those with Lr-0.17, Lr-0.18, or Lr-0.37. Furthermore, litter size at weaning of dams with Lr-0.23 was also significantly smaller than those dams with Lr-0.16, Lr-0.17, Lr-0.18, or Lr-0.37. The small litter size of dams with Lr-0.23 correlated with the smaller body sizes of these MMPs. These results suggest that SLA class II haplotypes are useful differential genetic markers for further haplotypic and epistatic studies of reproductive traits, selective breeding programs, and improvements in the production and reproduction performances of MMPs.

## 1. Introduction

A Microminipigs (MMP) is a miniature pig for laboratory use with an extremely small body size developed by Fuji Micra Inc. in Japan. The body sizes, such as body weight, height, chest width, and chest circumference at 4–6 months of age were much smaller than those of young adult beagles at 10 months old [1,2,3]. In the population of MMPs, eleven swine leukocyte antigen (SLA) class I and II high resolution haplotypes, including three recombinant haplotypes, were identified in the 14 parents of the offspring cohorts, and the dams and sires of the inbred MMP herd, since its serendipitous establishment in Japan in 2008 [4].

The major histocompatibility complex (MHC) region has been fully sequenced for the Large White pig breed with the haplotype Hp-1.1 at was found to be consist with class I, II, and III gene regions that characterize the core architecture and organization of most mammalian MHCs, including those of primates. A total of 151 loci were annotated within the 2.4-Mb sequences, including three classical (*SLA-1*, *SLA-2*, and *SLA-3*), and three non-classical class I genes (*SLA-6*, *SLA-7*, and *SLA-8*) in the class I region, and four classical (*DRA, DRB1*, *DQA*, and *DQB1*), and four non-classical class II genes (*DMA*, *DMB*, *DOA*, and *DOB*) in the class II region as expressed SLA genes [5]. Based on the genomic and cDNA sequences in the SLA loci, many kinds of molecular-based SLA typing systems, including polymerase chain reaction (PCR) - sequence-specific primers (SSPs) [6,7], -fluorescently labeled sequence-specific oligonucleotide probes (SSOPs) [8], and -sequence-based typing (SBT) by traditional Sanger methods and/or next generation sequencing (NGS) [4,9,10,11,12] have been reported to assign SLA class I and II alleles. A systematic nomenclature for the SLA genes, alleles, and haplotypes are established by the SLA Nomenclature Committee, formed in 2002. There are currently 237 class I, 211 class II alleles officially designated, and registered to the Immunopolymorphism Database–Major Histocompatibility Complex (IPD-MHC) SLA sequence database (www.ebi.ac.uk/ipd/mhc/group/SLA) Release 3.3.0.0 (2019-06-13) build 785; and 29 class I and 21 class II haplotypes have been defined by means of high-resolution DNA sequencing [13]. In addition, recently 10 class I and 12 class II novel haplotypes have been defined and designated by the SLA Nomenclature Committee (Ho et al. unpublished data).

The MHC class I and class II genes are highly polymorphic and have important roles in regulating the immune system against infectious diseases [14,15] and influencing various biological traits, such as immune recognition, autoimmunity, mating preferences, and pregnancy outcomes [16]. The polymorphisms of SLA genes have enabled the analysis of associations between genotypes, alleles, haplotypes, and various infections and phenotypes, including reproductive performance and production traits [17,18,19,20,21,22,23]. The genotypes or SNPs at one gene locus are often used to associate DNA sequence diversity with phenotypes or disease traits. However, the single locus analysis of variants as genotypes or alleles misses the epistatic or linked effects of variants at multiple loci on phenotypes and diseases. In this regard, haplotyping of heterozygous SNPs in genomic DNA was developed as a multi-locus method, in order to study the effect of different combination of genes on physiological and disease traits, and for elucidating population structure and histories [17,24,25,26]. Thus, genomic information, reported as haplotypes rather than isolated genotypes or SNPs, has become increasingly important in genomic medicine and for elucidating the links between SNPs, gene regulation, and protein function [24,27].

SLA-homozygotes with alleles at each of the class I and II genes (as homozygous haplotypes) can be obtained more commonly with the establishment of new inbred colonies, like the MMP population [4], than with those in outbred colonies with high heterozygosity, such as the Chinese Wuzhishan minipigs [28]. The polymorphic SLA haplotypes and SLA-homozygotes in the highly inbred MMP population are naturally phased, and they are often simpler to analyze than the highly outbred or heterozygote breeds with respect to the comparison of the effects among SLA haplotypes on various genetic traits. For example, when comparing birth weights and weights on postnatal day 50 among eight SLA class II haplotypes in the MMP population, both of the weights in piglets, the SLA class II low resolution haplotype (Lr)-0.23 were significantly lower than those in piglets with Lr-0.17 or Lr-0.37 [3].

In humans, certain HLA antigens between couples in closely related populations may lead to infertility and miscarriages [29]. Many studies showed that the sharing of certain maternal-fetal or paternal HLA and BoLA antigens may influence fetal development and survival [29,30]. Similarly, in pigs, the influence of SLA-encoded genes on reproductive performances has been reported as the association between specific SLA haplotypes and genital tract development in males, ovulation rates, litter size, and piglet weight at birth and weaning [3,17,18,19,20]. The SLA complex also appears to influence baby pig mortality [22]. However, in most of the pig studies, SLA haplotypes were assigned using serological typing methods. Recently, we analyzed the genetic association of the relationships between the performance of swine reproduction and SLA haplotypes, assigned by SLA-DNA typing techniques in other breeds of pigs [20,22]. In selective breeding Duroc pigs with two SLA class II haplotypes, Lr-0.13 and Lr-0.30, assigned by a PCR-SSP method, Lr-0.30 was associated with higher weaning and rearing rates [20]. In addition, in a Landrace pig line selected for resistance to mycoplasmal pneumonia, PCR-SBT and PCR-SSOP methods were used for assignments of 11 SLA-class II haplotypes, and the associations between haplotypes and immune-related traits and reproductive traits, such as phagocytic activities of lymphocytes, activities of the alternative pathway of compliment, body weights and a rate of daily gain were observed [22]. In the present study, to evaluate the contribution of SLA class II genes on reproductive traits in MMPs, we compared the reproductive performances on fertility index, gestation periods, litter sizes, and number of stillbirth among SLA class II haplotypes, including SLA-homozygous individuals.

## 2. Materials and Methods

### 2.1. Animals

In this study, pigs were bred as a MMP herd at Fuji Micra Inc. (Fujinomiya, Japan) from June, 2008 to February, 2017. In the herd, the records of 2,288-cumulative matings of MMPs consisting of 129 sows and 58 boars assigned to eight different SLA class II haplotypes were utilized for measurement of reproductive performances, such as fertility indices, gestation periods, litter sizes at birth and weaning, and numbers of stillbirths per delivery. This study was approved by the Animal Care and Use Committee of Gifu University (#17042, May 26, 2017). The care and use of the laboratory animals were conducted in compliance with the guidelines of Good Laboratory Practice of Gifu University and Fuji Micra Inc.

### 2.2. SLA Class II Typing

SLA class II-DRB1 and DQB1 alleles were assigned by low-resolution SLA genotyping in 187 MMPs, using a PCR-SSP method, as described previously [4]. Eight types of low-resolution SLA class II haplotypes, Lr-0.7, Lr-0.11, Lr-0.13, Lr-0.16, Lr-0.17, Lr-0.18, Lr-0.23, and Lr-0.37 were determined by an analysis of the inheritance and segregation of eight and four alleles of the DRB1 and DQB1 genes, respectively, in descendants of the MMP population (Table 1). The expected high-resolution allele specificities in Table 1 are based on the 14 high resolution genotyped parents [4] of the offspring cohorts and the dams and sires of the inbred MMP herd in this study (Table 2). Consequently, we only needed to use the cheaper and more convenient low-resolution typing method to group the 187 individual MMP into their high resolution haplotypic groups for statistical comparisons and analysis.

### 2.3. Measurement of Reproductive Performances

The fertility index was calculated as the ratio of the number of deliveries to that of matings. The indices were calculated from the data on a total number of 2288 cumulative matings in 129 dams and 58 sires associated with eight kinds of SLA class II haplotypes. Gestation periods were measured for 1380 deliveries (2760 haplotypes) that represented an overall fertility index (1380/2288) of 60.3%. To eliminate the data on premature deliveries, the gestation periods were analyzed for continuous deliveries over 100 days after copulations. Litter sizes were measured at birth including the total number of living and stillbirth newborn piglets in 1402 deliveries and at weaning in 1384 deliveries. Abnormal piglet productions calculated as the number of stillbirths per delivery were analyzed for 1402 deliveries. To separate the influence of maternal and paternal SLA class II haplotypes on reproductive performances, the analyses of gestation periods, litter sizes at birth and weaning, and the number of stillbirth per delivery of matings were analyzed separately in dams or sires with homozygous or heterozygous haplotypes. These four reproductive performances were also analyzed in dams with homozygous haplotypes, Lr-0.11, Lr-0.17, Lr-0.23, or Lr-0.37 after 75, 78, 74, or 78 deliveries, respectively; and in sires with homozygous haplotypes, Lr-0.11, Lr-0.16, Lr-0.18, or Lr-0.23 after 141, 148, 142, or 148 deliveries, respectively (Table 2).

### 2.4. Statistical Analyses

Statistical comparisons were carried out by multiple group comparison with ANOVA and/or Kruskal-Walis and Sheffe’s F tests (BellCurve in Excel, Social Survey Research Information Co., Ltd. Tokyo, Japan). Pairwise comparisons were adjusted for multiple tests with a Bonferroni correction. Fertility indices were evaluated by the Chi-square for independence test, using an m × n contingency table. Data are indicated as means ± standard error, and p-values of less than 0.05 were considered significant.

## 3. Results

### 3.1. Reproductive Performance in MMPs

The most frequently observed haplotype in 129 sows and 58 boars was Lr-0.23 (30.6%, and 36.2%, respectively), followed by Lr-0.37 (23.6%, and 24.1%, respectively) and Lr-0.17 (17.1%, and 15.5%, respectively). The two least frequent haplotypes were Lr-0.7 (0.8% in sows, 0.9% in boars) and Lr-0.13 (5.0% in sows, 1.7% in sires) (Table 1). Data of reproductive performance in MMPs with the lowest frequency haplotype, Lr-0.7, were excluded from all of the statistical analyses. A total of 1410 pregnancies were obtained as the result of 2,288 matings of 187 MMPs, representing a fertility index of 61.6%. The fertility index in MMPs was considerably lower than 88.4 ± 4.6 (standard deviation (SD)) in mixed breed domestic pigs in Japan [31]. Of the other reproductive performances in MMPs, the mean values of gestation period, while litter sizes at birth and weaning were 115.1 days, and 5.5 and 3.9 piglets/delivery, respectively. The gestation period in MMPs was comparable with those in other pig breeds [18,32,33]. Litter sizes at birth and weaning in MMPs were smaller than those in domestic pigs; 10.6 in commercial mixed breed pig herds in Japanese farm [34], 11.05 ± 0.77 (SD) in mixed breed domestic pigs in Japan [35], and 8.0—8.4 in Iberian pigs [36]. On the other hand, the litter size at birth in Göttingen minipigs was 5 to 6 piglets, which was slightly larger than that in MMPs [37]. However, the litter sizes in MMPs were slightly larger than that in other miniature pigs, NIBS minipigs; 4.4 ± 1.5 (SD) and 3.4 ± 1.3 (SD) piglets at birth and weaning, respectively [38], even though body sizes of the MMPs were considerably smaller than the NIBS minipigs produced in Japan [1,2] (Table 2).

### 3.2. Association between SLA Class II Haplotypes and Fertility Index

**Figure 1:**
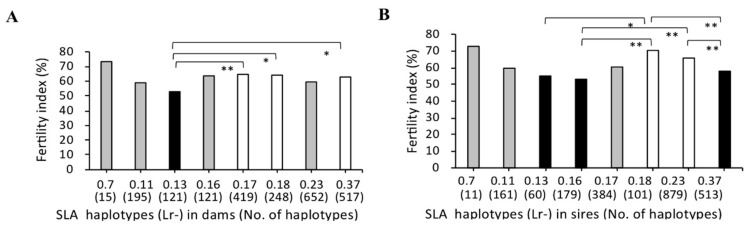
Comparison of the fertility indices of microminipigs (MMP) with different swine leukocyte antigen (SLA) class II haplotypes. *X*-axis shows the haplotypes of homozygous or heterozygous dams (**A**) and sires (**B**) and number of matings for each haplotype in brackets (no. of haplotypes). The *Y*-axis shows the fertility index as indicated by the ratio (%) of the number of deliveries to the number of matings, expressed as the mean value (bar). The number of haplotypes was counted as two in homozygous individuals. Black and white bars represent lower, and higher fertility indices, respectively, of the mean values, and the significant differences among haplotypes are indicated by the asterisks. Probabilities of significant differences among haplotypes are indicated by single (*p* < 0.05) and double (*p* < 0.01) asterisks. Gray bars represent mean values of fertility indices without significant differences among the haplotypes.

**Figure 2:**
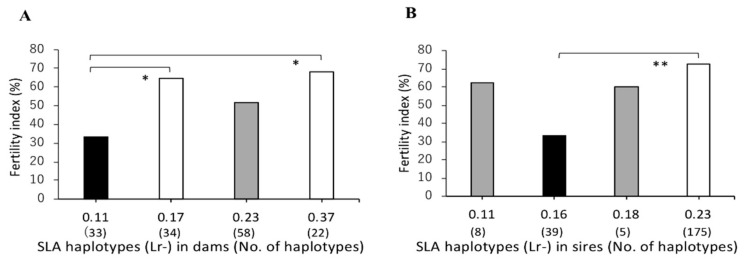
Comparison of fertility indices among SLA class II haplotypes in homozygous MMPs. *X*-axis shows the haplotypes of homozygous dams (**A**) and sires (**B**) and the number of matings for each haplotype in brackets (no. of haplotypes). The *Y*-axis shows the fertility index as indicated by the ratio (%) of the number of deliveries to the number of matings, expressed as the mean value (bar). Black and white bars represent lower, and higher fertility indices, respectively, of the mean values and the significant differences among haplotypes are indicated by the asterisks. Probabilities of significant differences among haplotypes are indicated by single (*p* < 0.05) and double (*p* < 0.01) asterisks. Gray bars represent the mean values of the fertility indices without significant differences among the haplotypes.

The mean value of the fertility index of each SLA haplotype with dams and sires were distributed across relatively wide ranges, from 52.9–73.3%, and 53.1–72.7%, respectively. The mean value of fertility index of dams was significantly lower for those with Lr-0.13 than those with Lr-0.17, Lr-0.18, or Lr-0.37 (*p* < 0.05, Figure 1A). Furthermore, the mean value of fertility index of boars with Lr-0.13 as mating partners of sows also was significantly lower than those with Lr-0.18 (*p* < 0.05, Figure 1B). Moreover, the mean values of fertility indices in mating with sires carrying Lr-0.16 or Lr-0.37 were significantly lower than those of sires carrying Lr-0.18 or Lr-0.23. Relatively high mean values of fertility indices, 73.3% and 72.7%, were observed in dams and sires as mating partners of sows with Lr-0.7, respectively. Furthermore, homozygous dams with Lr-0.11 had significantly lower fertility index than Lr-0.17 or Lr-0.37 (*p* < 0.05; Figure 2A). In contrast, homozygous sires with Lr-0.16 had significantly lower fertility index than those with Lr-0.23 (*p* < 0.01; Figure 2B).

### 3.3. Association of SLA Class II Haplotypes and Gestation Periods

**Figure 3:**
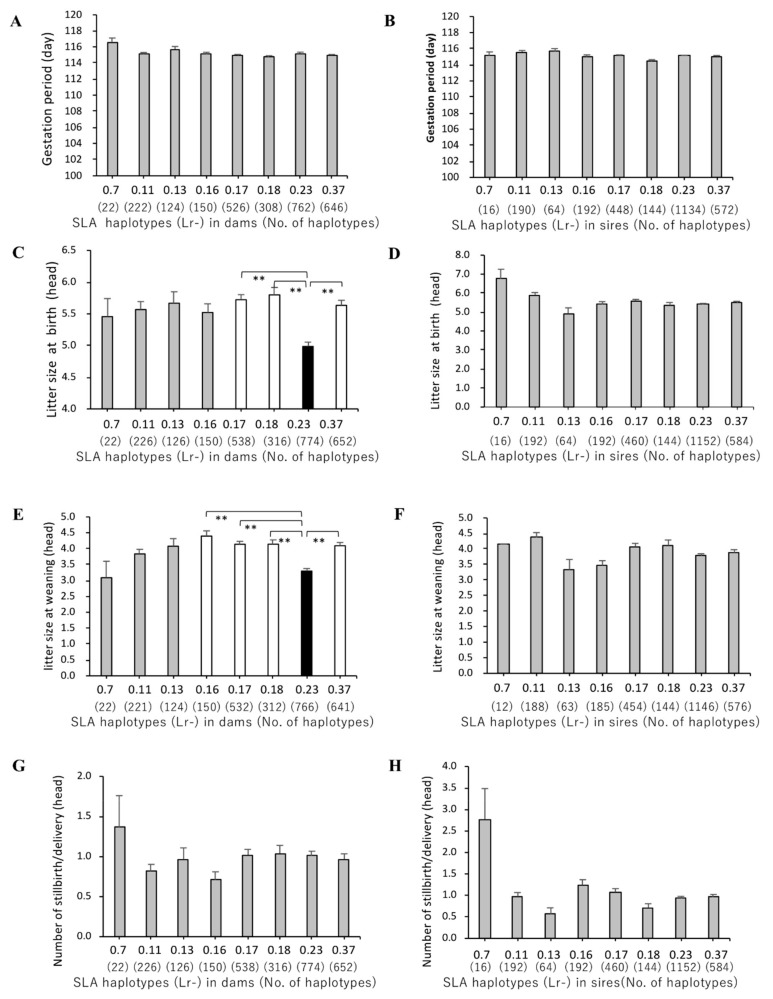
Comparison of reproductive performances of MMPs with different SLA class II haplotypes. *X*-axis for A to H shows the homozygous or heterozygous SLA haplotypes and the number of matings for each haplotype in brackets (no. of haplotypes). Each bar along the *X*-axis indicates the mean values and whiskers (standard errors) of gestation periods in dams (**A**) and sire (**B**), litter sizes at birth in dams (**C**) and sires (**D**), litter sizes at weaning in dams (**E**) and sires (**F**), and the number of stillbirths/delivery in dams (**G**) and sires (**H**) shown along the *Y*-axis. Black and white bars represent lower and higher haplotypes, respectively, with mean values of each trait and significant differences among haplotypes. The gray bars represent mean values of haplotypes without any significant differences among the haplotypes. The probabilities of significant differences among haplotypes are indicated by single (*p* < 0.05) and double (*p* < 0.01) asterisks.

**Figure 4:**
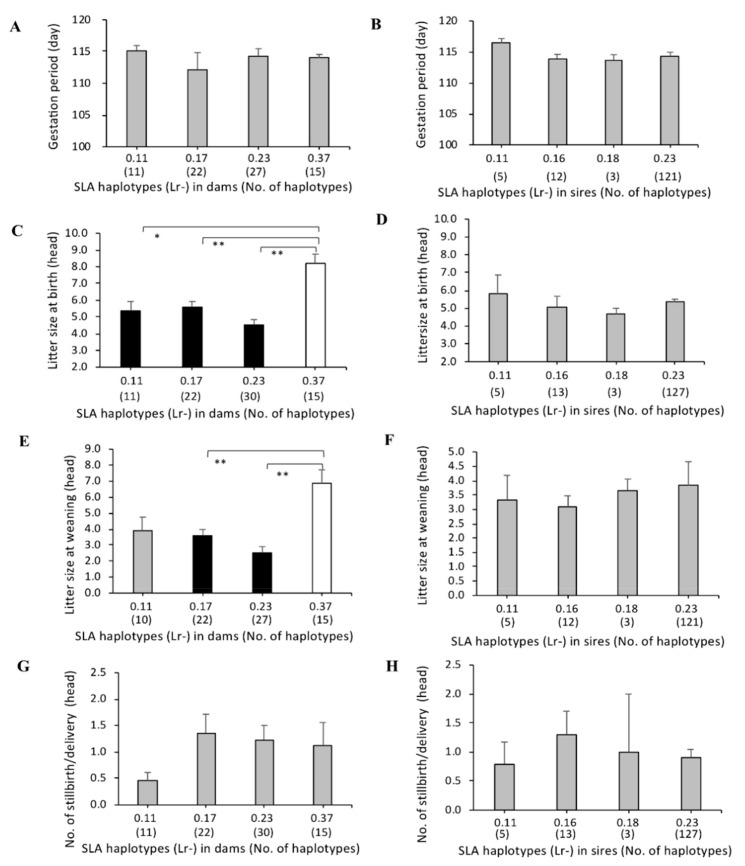
Comparison of reproductive performances among SLA class II homozygous haplotypes in MMPs. Haplotypes and number of deliveries for each haplotype are shown along the *X*-axis, and the bars for each homozygous haplotype show the mean values and whiskers (standard errors) of gestation periods in dams (**A**) and sire (**B**), litter sizes at birth in dams (**C**) and sires (**D**), litter sizes at weaning in dams (**E**) and sires (**F**), and the number of stillbirths/delivery in dams (**G**) and sires (**H**) along the *Y*-axis. Black and white bars represent lower and higher haplotypes, respectively, and mean values of each trait with significant differences among haplotypes. Gray bars represent the mean values of haplotypes without any significant differences among the haplotypes. The probabilities of significant differences among haplotypes are indicated by single (*p* < 0.05) and double (*p* < 0.01) asterisks.

The mean values of gestation periods were within the small ranges of 115.0–116.6 or 113.8–115.6 among the eight SLA class II haplotypes for dams or sires, used as mating partners of sows, respectively, in total number of 1380 deliveries. There were no significant differences between the mean values of gestation periods among the haplotypes (Figure 3A,B). In addition, no obvious differences were observed between the mean values for gestation periods of four homozygous haplotypes, Lr-0.11, Lr-0.17, Lr-0.23, and Lr-0.37 in dams, and Lr-0.11, Lr-0.16, Lr-0.18 and Lr-0.23 in sires (Figure 4A,B). These results suggest that there are no maternal or paternal SLA class II genotype effects on gestation periods.

### 3.4. Association between SLA Class II Haplotypes and Litter Sizes at Birth and Weaning

The dams with Lr-0.23 had the smallest litter sizes at birth compared to those with other SLA class II haplotypes. The mean value of litter sizes at birth for dams with Lr-0.23 was significantly smaller than those with Lr-0.17, Lr-0.18 or Lr-0.37 (*p* < 0.01; Figure 3C) and Lr-0.11 (*p* < 0.05; Figure 3C). In contrast, no significant differences on litter sizes at birth were detected among sires with seven SLA class II haplotypes except Lr-0.7 suggesting that there are no effects of paternal SLA types on the trait (Figure 3D). In the dams with Lr-0.23, the mean value of litter sizes at weaning rates was also significantly smaller than those with Lr-0.16, Lr-0.17, Lr-0.18, or Lr-0.37 (*p* < 0.01; Figure 3E). Moreover, the mean litter sizes at birth and weaning of piglets of dams with the homozygous Lr-0.23 haplotypes were smaller than those of dams with homozygous Lr-0.11, Lr-0.17, or Lr-0.37. Furthermore, dams with homozygous Lr-23 had significantly smaller litter sizes at birth and weaning than homozygous Lr-0.37 (*p* < 0.01; Figure 3C,E). In contrast, there were no significant differences between the mean values of litter sizes at birth and weaning among the seven haplotypes in sires (Figure 3D,F). Furthermore, there were no significant effects of SLA class II haplotypes on litter sizes in sires with homozygous Lr-0.11, Lr-0.16, Lr-0.18, or Lr-0.23 (Figure 4D,F).

### 3.5. Association of SLA Class II Haplotypes and Number of Stillbirths per Delivery

The largest mean values of the number of stillbirths per deliveries were observed for both the dams and sires with Lr-0.7 (Figure 3G,H). Homozygous pigs with Lr-0.7 as mating pairs could not be found in the 129 dams and fifty-eight sires. Due to the low frequencies (0.8% in dams, 0.9% in sires) of MMPs with Lr-0.7, no statistical comparisons of the mean numbers of stillbirths per delivery between Lr-0.7 and the other seven haplotypes were carried out. However, statistical analysis of these seven haplotypes showed that there were no significant differences among them for the mean values of the number of stillbirths per deliveries. Also, no significant differences among the mean numbers of stillbirths per delivery were observed in dams with homozygous Lr-0.11, Lr-0.17, Lr-0.23 or Lr-0.37, and sires with homozygous Lr-0.11, Lr-0.16, Lr-0.18 or Lr-0.23 (Figure 4G,H).

## 4. Discussion

To improve reproduction performances using genetic marker-assisted selection, reproductive traits, such as gestation periods and litter sizes have been analyzed in the pig populations of various breeds, including the MMPs with the extra small body sizes [31,32,33,34,35,36,37,38]. In comparing reproductive performances between the MMPs and other breeds of pigs [18,31,32,33,34,35,36,37,38], the mean gestation period, and litter sizes at birth and weaning in MMPs (Table 2) were similar to those of other breeds of domestic pigs [18,31,32], including Göttingen and NIBS minipigs [37,38]. Thus, the inbred MMP population, used in the present study, showed relatively normal reproductive traits that were useful and easier for evaluating the differential effects of SLA homozygous and heterozygous haplotypes on reproductive traits, than by using the more confounding SLA heterozygous haplotypes of most other breeds.

In this study, we haplotyped SLA class II alleles using a low resolution PCR-SSP method in well-defined MMP segregating families, consisting of 187 MMPs, and examined the association between the haplotypes and various reproduction traits, such as fertility indices, gestation periods, litter sizes at birth and weaning, and the numbers of stillbirths per delivery. The off-spring were segregated into the SLA class II low resolution haplotype families, based on the genotype results of the 129 sows and 58 boars that were assigned to eight different SLA class II haplotypes (Table 1). Moreover, we estimated the expected high-resolution allele specificities of the eight different SLA class II haplotypes (Table 1) because they could be easily inferred from the 14 high resolution genotyped parents [4] of all the offspring cohorts, and the dams and sires of the inbred MMP herd in this study (Table 2). Consequently, because of the stability of ancestral haplotypes due to low mutation rates in mammals [25], we only needed to use the much cheaper and more convenient low-resolution typing methods to group the 187 individual MMP into their low resolution (or inferred high resolution) haplotypic groups for statistical comparisons and analysis.

The significant effects of two SLA class II haplotypes, Lr-0.13 and Lr-0.18, in both dams and sires showed lower, and higher fertility indices, respectively. In our present data, we could not determine whether much lower or higher fertility indices would be observed in mating pairs with homozygous haplotypes Lr-0.13 or Lr-0.18, respectively. Dams with Lr-0.17, Lr-0.18, or Lr-0.37 tended to have a relatively high fertility index, litter sizes at birth and weaning, and low number of stillbirths per delivery, suggesting relatively good performances of reproduction traits. In general, these haplotypes were at relatively high frequency in the MMP population (Table 1). Moreover, both sires and dams with the Lr-0.18 haplotype showed similar trends for the four reproduction traits. In contrast, sires and dams exhibited an opposite trend for associations between Lr-37 and fertility index; sires with Lr-0.37 had relatively low fertility index suggesting that the contribution of Lr-0.37 on fertility may be different between sows and boars in a MMP herd. On the other hand, the large numbers of stillbirths per delivery observed in sires with Lr-0.7 are consistent with the production of low frequencies of pigs with this haplotype in the MMP population (Table 1).

The Lr-0.23 haplotype has the highest frequency of 36.2% in dams of the MMP population (Table 1), and they also had the smallest litter size at birth compared to the dams with the other seven haplotypes (Figure 3C). Furthermore, litter sizes at weaning of the dams with Lr-0.23 were significantly smaller than those of dams with Lr-0.16, Lr-0.17, Lr-0.18, or Lr-0.37. In our previous analyses of association between SLA haplotypes and body weights in MMPs, piglets with the Lr-0.23 had lower body weights at birth (0.415 kg) and on postnatal day 50 (3.14 kg) than those with the other SLA haplotypes, although they had no significant differences in daily gains (DGs) in comparison to those with the other haplotypes [3]. These data on low body weights and DGs of MMPs with Lr-0.23 characterize their small body size and slow growth rates; although their small litter size at weaning might simply reflect their small size at birth. Nevertheless, in the MMP population, Lr-0.23 is one of the genetic markers both for small body size and small litter size. Taken together, the associations between specific SLA haplotypes and reproduction traits showed that SLA class II haplotypes differ among the various traits of reproduction performance in MMPs, and they might be useful genetic markers for the improvement of production and reproduction performances in selective breeding programs.

A large litter size is commonly used as a measure of successful breeding and animal production [39,40]. Although animal production management strives for large litter sizes, there can be serious limitations and biological problems with large litter sizes, such as increased numbers of stillbirths. In some cases, moderately low litter sizes can be of greater benefit to the overall animal production than large litter sizes and benefits, such as greater lactation efficiency for the mother and lower nutritional requirements for the litter and the mother [40]. There are many biological and genetic factors involved in litter size and/or embryonic and foetal growth rates [39,41,42]. Here, we focused on the effect of MHC class II haplotypes on reproductive traits and found that the embryonic survival and litter size increases with the probability that the embryo receives the haplotype from the dam, rather than the boar. In this regard, the MHC-DRB1 and -DQB1 genes expressed by placental macrophages [43,44] might affect embryonic growth rates and litter size as a consequence of gene activations under various regulatory scenarios, such as methylation or gene activation due to stress or infections. An alternative explanation for the correlation between MHC class II haplotypes and litter size is the haplotypic linkage between MHC class II alleles and various other gene alleles located within and outside the SLA region. The linkage between the MHC class I and class II alleles was not investigated in this study, but MHC class I is known to mediate immunological tolerance, leucocyte recruitment, and the development of the trophoblast during pregnancy, and rejection during parturition in the bovine [30]. The MHC also has effects on placental leucocyte recruitment during gestation and parturition in the mouse [44]. There are numerous other genes within the MHC class III or extended class II region that have a possible role in the reproduction and lactation processes. For example, butyrophilin is involved genetically and biologically with lactation [45], RING1 with polycomb function during oogenesis [46], KIFC1 with oocyte meiosis [47], and POU1F5 (OCT4) transcription factor in stem cell modulation and embryonic development [48].

The polymorphic MHC class II haplotypes also might be linked with one or other of the SNPs of 376 functional genes outside the MHC region that were associated significantly with reproductive traits in Large White pigs [49]. Multiple genes were associated with some swine production traits and many of them were mapped on several chromosomes including *Sus scrofa* chromosome (SSC) 7 (Animal Quantitative Trait Loci (QTL) Database, https://www.animalgenome.org/QTLdb). QTL detection analyses for traits on growth and fatness indicated that the SLA region on SSC7 was excluded as a candidate region [50]. However, in a herd of Meishan/Large White pigs, Wei et al. mapped QTL influencing growth traits near the SLA region [51]. Taken together, these reports suggest that the SLA genes or haplotypes associate indirectly with those traits. Nevertheless, to better define the SLA or other loci within the SLA region that are responsible for productive and reproductive traits, further association analyses will need to be carried out using next generation sequencing (NGS) techniques in MMPs.

For a few decades, several authors reported that SLA haplotypes are associated with various traits or measures of growth and reproduction performances, suggesting the possibility of SLA molecules as important determinants on many economical traits [17,18,19,52]. Because it is difficult to define the most likely corresponding locus for productive or reproductive performance within the SLA region simply by the data analyses of serologically assigned SLA haplotypes. Many different molecular-based SLA typing techniques were developed and used for the analyses of associations of production and reproduction traits and SLA alleles or haplotypes [3,22,23]. These association data often indicated that SLA alleles or haplotypes are useful genetic markers to achieve improvements in pig breeding programs. These studies also highlighted the advantage of using well-defined, genetically conserved haplotypes over just using trait-associated SNPs for linking the MHC with reproduction, and possibly other medical and phenotypic traits of interest. Since our association studies were analyzed using only a few SLA haplotypic class II loci in a limited pig population and breed, similar haplotype studies will need to be expanded to other MHC loci and other breeds, populations and species for comparison and confirmation that the MHC haplotypic markers might be associated productively with animal breeding performance.

## 5. Conclusions

In our study of the unique inbred MMP population, association analyses between specific SLA haplotypes and reproduction traits exhibited significant effects of SLA class II homozygous haplotypes on reproduction performances, that differed among fertility index, litter sizes, and number of stillbirths. The present study forms a basis for a broader and more detailed investigation of the differential effects of the MHC class I, II and III genes as well as the many other genes outside the MHC complex that are known to influence reproduction in pigs and various other mammalian species.

## Figures and Tables

**Table 1:** SLA-class II genotypes and number of SLA class II haplotypes in Microminipigs.

SLA Class II	Allele Specificity by Low Resolution Typing		* Expected Allele Specificity by High Resolution Typing		Number of Haplotypes (Frequency (%))			
haplotype	DRB1	DQB1	DRB1	DQB1	Dams		Sires	
Lr-0.7	06:XX	06:XX	06:01	06:01	2	(0.8)	1	(0.9)
Lr-0.11	09:XX	04:XX	09:01	04:01:02/04:02	19	(7.4)	10	(8.6)
Lr-0.13	04:XX	03:XX	04:03	03:03	13	(5.0)	2	(1.7)
Lr-0.16	11:XX	06:XX	11:03	06:01	16	(6.2)	7	(6.0)
Lr-0.17	08:XX	05:XX	08:01	05:01/05:02	44	(17.1)	18	(15.5)
Lr-0.18	14:XX	04:XX	14:01	04:01:02/04:02	24	(9.3)	8	(6.9)
Lr-0.23	10:XX	06:XX	10:01	06:01	79	(30.6)	42	(36.2)
Lr-0.37	07:XX	05:XX	07:01	05:01/05:02	61	(23.6)	28	(24.1)

Total number of dams and sires were 129 and 58, respectively. * Expected allele specificity by high resolution typing indicates deduced alleles by low resolution typing at two digit level in Microminipigs. DQB1*04:02 (Hp-0.7) and DQB1*04:01:02 (Hp-0.23), and DQB1*05:01 (Hp-0.17) and DQB1*05:02 (Hp-0.37) are assigned as DQB1*04XX (Lr-0.7 or Lr-0.23) and DQB1*05XX (Lr-0.17 or Lr-0.37) using a PCR-SSP method, respectively.

**Table 2:** Summary of reproduction traits in 187 Microminipigs.

Trait	Mean *	SE *	Number of Haplotypes **
Gestation period (day)	115.1	0.06	2760
Litter size at birth (No. of piglets)	5.48	0.04	2804
Litter size at weaning (No. of piglets)	3.88	0.04	2804
No. of stillbirth/delivery (No. of piglets)	0.97	0.03	2768

* Mean and SE indicate mean value and standard error, respectively. ** Number of haplotypes indicated as total number of haplotypes consisting of various mating combinations among 129 dams and 58 sires in each trait.

## References

[1] Kaneko N., Itoh K., Sugiyama A., Izumi Y. (2011). Microminipig, a non-rodent experimental animal optimized for life science research: Preface. J. Pharmacl. Sci..

[2] Takasu M., Tsuji E., Imaeda N., Kaneko N., Matsubara T., Maeda M., Ito Y., Shibata S., Ando A., Nishii N. (2015). Body and, major organ sizes of young mature Microminipigs determined by computed tomography. Lab. Anim..

[3] Matsubara T., Takasu M., Imaeda N., Nishii N., Takashima S., Nishimura T., Nishimura T., Shiina T., Ando A., Kitagawa H. (2018). Genetic association of swine leukocyte antigen class II haplotypes and body weight in Microminipigs. Asian Aust. J. Animal Sci..

[4] Ando A., Imaeda N., Ohshima S., Miyamoto A., Kaneko N., Takasu M., Shiina T., Kulski J.K., Inoko H., Kitagawa H. (2015). Characterization of swine leucocyte antigen alleles and haplotypes on a novel miniature pig line, Microminipig. Anim. Gen..

[5] Renard C., Hart E., Sehra H., Beasley H., Coggill P., Howe K., Harrow J., Gilbert J., Sims S., Rogers J. (2006). The genomic sequence and analysis of the swine major histocompatibility complex. Genomics.

[6] Ho C.S., Lunney J.K., Franzo-Romain M.H., Martens G.W., Lee Y.J., Lee J.H., Wysocki M., Rowland R.R., Smith D.M. (2009). Molecular characterization of swine leucocyte antigen class I genes in outbred pig populations. Anim. Genet..

[7] Ho C.S., Lunney J.K., Franzo-Romain M.H., Martens G.W., Rowland R.R., Smith D.M. (2010). Molecular characterization of swine leucocyte antigen class II genes in outbred pig populations. Anim. Genet..

[8] Ando A., Shigenari A., Ota M., Sada M., Kawata H., Azuma F., Kojima-Shibata C., Nakajoh M., Suzuki K., Uenishi H. (2011). SLA-DRB1 and -DQB1 genotyping by the PCR-SSOP-Luminex method. Tissue Antigens.

[9] Kita Y.F., Ando A., Tanaka K., Suzuki S., Ozaki Y., Uenishi H., Inoko H., Kulski J.K., Shiina T. (2012). Application of high-resolution, massively parallel pyrosequencing for estimation of haplotypes and gene expression levels of swine leukocyte antigen (SLA) class I genes. Immunogenetics.

[10] Le M., Choi H., Choi M.K., Cho H., Kim J.H., Seo H.G., Cha S.Y., Seo K., Dadi H., Park C. (2015). Development of a simultaneous high resolution typing method for three SLA class II genes, SLA-DQA, SLA-DQB1, and SLA-DRB1 and the analysis of SLA class II haplotypes. Gene.

[11] Sørensen M.R., Ilsøe M., Strube M.L., Bishop R., Erbs G., Hartmann S.B., Jungersen G. (2017). Sequence-Based Genotyping of Expressed Swine Leukocyte Antigen Class I Alleles by Next-Generation Sequencing Reveal Novel Swine Leukocyte Antigen Class I Haplotypes and Alleles in Belgian, Danish, and Kenyan Fattening Pigs and Göttingen Minipigs. Front. Immunol..

[12] Lee C., Moroldo M., Perdomo-Sabogal A., Mach N., Marthey S., Lecardonnel J., Wahlberg P., Chong A.Y., Estellé J., Ho S.Y.W. (2018). Inferring the evolution of the major histocompatibility complex of wild pigs and peccaries using hybridisation DNA capture-based sequencing. Immunogenetics.

[13] Ho C.S., Lunney J.K., Ando A., Rogel-Gaillard C., Lee J.H., Schook L.B., Smith D.M. (2009). Nomenclature for factors of the SLA system, update 2008. Tissue Antigens.

[14] Blackwell J.M., Jamieson S.E., Burgner D. (2009). HLA and infectious diseases. Clin. Microbiol. Rev..

[15] Gutiérrez S.E., Esteban E.N., Lützelschwab C.M., Juliarena M.A., Abubakar M. (2017). Chapter 6, Major Histocompatibility Complex-associated resistance to infectious diseases: The case of Bovine leukemia virus infection. Trends and Advances in Veterinary Genetics.

[16] Sommer S. (2005). The importance of immune gene variability (MHC) in evolutionary ecology and conservation. Front. Zool..

[17] Renard C., Vaiman M. (1989). Possible relationships between SLA and porcine reproduction. Reprod. Nutr. Dev..

[18] Gautschi C., Gaillard C. (1989). Influence of major histocompatibility complex on reproduction and production traits in swine. Anim. Genet..

[19] Conley A.J., Jung Y.C., Schwartz N.K., Warner C.M., Rothschild M.F., Ford S.P. (1988). Influence of SLA haplotype on ovulation rate and litter size in miniature pigs. J. Reprod. Fertil..

[20] Imaeda N., Ando A., Takasu M., Matsubara T., Nishii N., Takashima S., Shigenari A., Shiina T., Kitagawa H. (2018). Influences of swine leukocyte antigen haplotypes on serum antigen titers against swine erysipelas vaccine and traits of reproductive ability and meat production in a SLA-defined Duroc pigs. J. Vet. Med. Sci..

[21] Vaiman M., Chardon P., Rothschild M.F. (1998). Porcine major histocompatibility complex. Rev. Sci. Tech. Off. Int. Epiz..

[22] Ando A., Shigenari A., Kojima-Shibata C., Nakajoh M., Suzuki K., Kitagawa H., Shiina T., Inoko H., Uenishi H. (2016). Association of swine leukocyte antigen class II haplotypes and immune-related traits in a swine line selected for resistance to mycoplasmal pneumonia. Comp. Immunol. Microbiol. Infect. Dis..

[23] Zhang S., Yang J., Wang L., Li Z., Pang P., Li F. (2018). SLA-11 mutations are associated with litter size traits in Large White and Chinese DIV pigs. Anim. Biotech..

[24] Glusman G., Cox H.C., Roach J.C. (2014). Whole-genome haplotyping approaches and genomic medicine. Genome Med..

[25] Sánchez-Molano E., Tsiokos D., Chatziplis D., Jorjani H., Degano L., Diaz C., Rossoni A., Schwarzenbacher H., Seefried F., Varona L. (2016). A practical approach to detect ancestral haplotypes in livestock populations. BMC Genet..

[26] Huang M., Tu J., Lu Z. (2017). Recent Advances in Experimental Whole Genome Haplotyping Methods. Int. J. Mol. Sci..

[27] Murphy N.M., Burton M., Powell D.R., Rossello F.J., Cooper D., Chopra A., Hsieh M.J., Sayer D.C., Gordon L., Pertile M.D. (2016). Haplotyping the human leukocyte antigen system from single chromosomes. Sci. Rep..

[28] Min F., Pan J., Wang X., Chen R., Wang F., Luo S., Ye J. (2014). Biological characteristics of captive Chinese Wuzhishan minipigs (*Sus scrofa*). Int. Sch. Res. Notices.

[29] Agenor A., Bhattacharya S. (2015). Infertility and miscarriage: Common pathways in manifestation and management. Womens Health.

[30] Rapacz-Leonard A., Dąbrowska M., Janowski T. (2014). Major histocompatibility complex I mediates immunological tolerance of the trophoblast during pregnancy and may mediate rejection during parturition. Mediat. Inflamm..

[31] Suzuki K., Somei H. (1982). Present condition and opinions of artificial insemination in pig farming. Bull. Chiba Prefect. Livestock Exptl. Station.

[32] Rothkötter H.J., Sowa E., Pabst R. (2002). The pig as a model of developmental immunology. Hum. Exp. Toxicol..

[33] Tsumagari S., Mori J., Kanagawa H., Hamana K. (2002). Textbook of Theriogenology.

[34] Koketsu Y. (2002). Reproductive productivity measurements in Japanese swine breeding herds. J. Vet. Med. Sci..

[35] Yamane I., Ishizaki S., Yamazaki H. (2014). Parameters Contributing to Improved Reproductive Performance at Farrow-to-Finish Swine Farms in Japan. J. Jpn. Vet. Med. Assoc..

[36] Casellas J., Ibáñez-Escriche N., Varona L., Rosas J.P., Noguera J.I. (2019). Inbreeding depression load for litter size in *Entrepelado* and *Retinto* Iberian pig varieties. J. Anim. Sci..

[37] Schuleri K.H., Boyle A.J., Centola M., Amado L.C., Evers R., Zimmet J.M., Evers K.S., Ostbye K.M., Scorpio D.G., Hare J.M. (2008). The adult Göttingen Minipig as a model for chronic heart failure after myocardial infarction: Focus on cardiovascular imaging and regenerative therapies. Comp. Med..

[38] Saitoh T. (2009). Utilization of a swine other than food. All About Swine.

[39] Lawlor P.G., Lynch P.B. (2007). A review of factors influencing litter size in Irish sows. Ir. Vet. J..

[40] Rutherford K.M.D., Baxter E.M., D’Eath R.B., Turner S.P., Arnott G., Roehe R., Ask B., Sandoe P., Moustsen V.A., Thorup F. (2013). The welfare implications of large litter size in the domestic pig I: Biological factors. Anim. Welfare.

[41] Chen P., Baas T.J., Mabry J.W., Koehler K.J., Dekkers J.C. (2003). Genetic parameters and trends for litter traits in U.S.; Yorkshire, Duroc, Hampshire, and Landrace pigs. J. Anim. Sci..

[42] Kwon S.G., Hwang J.H., Park D.H., Kim T.W., Kang D.G., Kang K.H., Kim I.S., Park H.C., Na C.S., Ha J. (2016). Identification of differentially expressed genes associated with litter size in Berkshire pig placenta. PLoS ONE.

[43] Athanassakis-Vassiliadis I., Thanos D., Papamatheakis J. (1989). Induction of class II major histocompatibility complex antigens in murine placenta by 5-azacytidine and interferon-γ involves different cell populations. Eur. J. Immunol..

[44] Kieckbusch J., Balmas E., Hawkes D.A., Colucci F. (2015). Disrupted PI3K p110δ signaling dysregulates maternal immune cells and increases fetal mortality in mice. Cell Rep..

[45] Stefferl A., Schubart A., Storch M., Amini A., Mather I., Lassmann H., Linington C. (2000). Butyrophilin, a milk protein, modulates the encephalitogenic T cell response to myelin oligodendrocyte glycoprotein in experimental autoimmune encephalomyelitis. J. Immunol..

[46] Posfai E., Kunzmann R., Brochard V., Salvaing J., Cabuy E., Roloff T.C., Liu Z., Tardat M., van Lohuizen M., Vidal M. (2012). Polycomb function during oogenesis is required for mouse embryonic development. Genes Dev..

[47] Camlin N.J., McLaughlin E.A., Holt J.E. (2017). Motoring through: The role of kinesin superfamily proteins in female meiosis. Hum. Reprod. Update.

[48] Wu G., Schöler H.R. (2014). Role of Oct4 in the early embryo development. Cell Regen..

[49] Wang Y., Ding X., Tan Z., Xing K., Yang T., Wang Y., Sun D., Wang C. (2018). Genome-wide association study for reproductive traits in a Large White pig population. Anim. Genet..

[50] Demeure O., Sanchez M.P., Riquet J., Iannuccelli N., Demars J., Fève K., Kernaleguen L., Gogué J., Billon Y., Caritez J.C. (2005). Exclusion of the swine leukocyte antigens as candidate region and reduction of the position interval for the *Sus scrofa* chromosome 7 QTL affecting growth and fatness. J. Anim. Sci..

[51] Wei W.H., Skinner T.M., Anderson J.A., Southwood O.I., Plastow G., Archibald A.L., Haley C.S. (2011). Mapping QTL in the porcine MHC region affecting fatness and growth traits in a Meishan/Large White composite population. Anim. Genet..

[52] Lunney J.K., Ho C.S., Wysocki M., Smith D.M. (2009). Molecular genetics of the swine major histocompatibility complex, the SLA complex. Dev. Comp. Immunol..

